# Sensitivity and Specificity Improvement in Abdominal Obesity Diagnosis Using Cluster Analysis during Waist Circumference Cut-Off Point Selection

**DOI:** 10.1155/2015/750265

**Published:** 2015-04-05

**Authors:** Valmore Bermúdez, Joselyn Rojas, Juan Salazar, Roberto Añez, Alexandra Toledo, Luis Bello, Vanessa Apruzzese, Robys González, Maricarmen Chacín, Mayela Cabrera, Clímaco Cano, Manuel Velasco, José López-Miranda

**Affiliations:** ^1^Endocrine and Metabolic Diseases Research Center, The University of Zulia, 20th Avenue, Maracaibo 4004, Venezuela; ^2^Clinical Pharmacology Unit, José María Vargas School of Medicine, Central University of Venezuela, Caracas 1051, Venezuela; ^3^Lipid and Atherosclerosis Unit, Department of Medicine, Carlos III Institute of Health, IMIBIC/Reina Sofia University Hospital/University of Córdoba and CIBER Obesity and Nutrition Physiopathology (CIBEROBN), 230002 Córdoba, Spain

## Abstract

*Introduction*. The purpose of this study was to analyze the influence of metabolic phenotypes during the construction of ROC curves for waist circumference (WC) cutpoint selection. *Materials and Methods*. A total of 1,902 subjects of both genders were selected from the Maracaibo City Metabolic Syndrome Prevalence Study database. Two-Step Cluster Analysis (TSCA) was applied to select metabolically healthy and sick men and women. ROC curves were constructed to determine WC cutoff points by gender. *Results*. Through TSCA, metabolic phenotype predictive variables were selected: HOMA2-IR and HOMA2-*β*cell for women and HOMA2-IR, HOMA2-*β*cell, and TAG for men. Subjects were classified as healthy normal weight, metabolically obese normal weight, healthy and metabolically disturbed overweight, and healthy and metabolically disturbed obese. Final WC cutpoints were 91.50 cm for women (93.4% sensitivity, 93.7% specificity) and 98.15 cm for men (96% sensitivity, 99.5% specificity). *Conclusions*. TSCA in the selection of the groups used in ROC curves construction proved to be an important tool, aiding in the detection of MOWN and MHO which cannot be identified with WC alone. The resulting WC cutpoints were <91.00 cm for women and <98.00 cm for men. Furthermore, anthropometry is insufficient to determine healthiness, and, biochemical analysis is needed to properly filter subjects during classification.

## 1. Introduction 

Obesity is emerging as an important health issue in Venezuela, particularly in urban areas, paradoxically coexisting with undernutrition [1]. The rising prevalence of overweight and obesity around the world shares a direct correlation with the increasing occurrence of obesity-related comorbidities such as high blood pressure (HBP), metabolic syndrome (MS), dyslipidemia, type 2 diabetes mellitus (T2DM), and cardiovascular disease (CVD) [2]. Several pathophysiological aspects have been proposed to explain the close relationship between these diseases, including the degree of adiposity and anatomic fat localization [1–3].

Currently, it is accepted that the majority of individuals who have obesity progressively develop insulin resistance, beta cell failure, and lastly T2DM, proving to be a biological continuum that is undeniably complicated and intricate [2, 3]. However, approximately 10–25% of obese individuals are metabolically healthy, most likely due to preserved insulin sensitivity probably due to genetic factors [4]. On the other hand, visceral adipose tissue inflammation, ectopic fat deposition, and adipose tissue dysfunction have been proposed as an etiologic triumvirate that mediates insulin resistance in human obesity independently of total body fat mass [3]. Furthermore, it has been reported that around 10–15% of lean subjects may exhibit insulin resistance and other metabolic disturbances like dyslipidemia, dysglycemia, and HBP [5]. This landscape suggests four well-defined phenotypes existence in human beings according to body composition and metabolic status: (a) healthy normal weight (HNW), (b) metabolically obese normal weight (MONW), (c) metabolically disturbed obese (MDO), and (d) metabolically healthy obese (MHO) [6–8].

It has been highlighted that the proposed waist circumference (WC) cut-off points for Latin America, as well as other parts of the world, have relatively low areas under the curve (AUC) and therefore relatively low sensitivities and specificities during COR curves construction [9, 10] when using traditional criteria to classify subjects as healthy or sick, such as the presence of two or more components of the MS criteria as reported by Hara et al. [11]. Recently, our group built ROC curves for WC cutoff-point selection using 2 or more positive MS components to differentiate between healthy and sick individuals, rendering values of 90.25 cm (68.4% sensitivity, 65.8% specificity) for women and 95.15 cm for men (71.1% sensitivity, 67.4% specificity) [12].

Nevertheless, it has been suggested that unusual metabolic phenotypes such as MONW and MHO could influence the accuracy of obesity-centered studies due to difficulties in subject characterization [6–8], and this setback includes sensitivity and specificity from WC cut-offs point selection for obesity diagnosis. The biological traits of these uncommon phenotypes [6–8, 13] result in uncharacteristic grouping of metabolic components which could be difficult to predict. Therefore, the* proof-of-concept* would be that the early detection of these phenotypes prior to WC selection could improve the accuracy of selected WC cutpoints, and their future application in epidemiological studies.

The possibility of detecting MONW and MHO prior to any cut-off point selection method cannot rely on common markers such as WC because they can be misleading, due to the uniqueness of such phenotypes [7, 8]. In this context, the advantage of applying data mining techniques (like Cluster analysis) is that it allows the spontaneous grouping of individuals according to the behavior of metabolic and anthropometric variables, superseding the discriminating capacity of internationally appointed WC cut-off points and other preestablished criteria for metabolic alterations. Since these phenotypes do not behave in the same manner as the common ones, they could be considered as “*noise*” during the construction of ROC curves and might affect the sensitivity of specificity of the selected cutpoints for WC. Thus, the ability to identify and filter them from the ROC construction process is not only optional, but actually necessary.

Taking all this information into consideration, the purpose of this investigation was to identify subjects with unusual metabolic phenotypes and, afterwards, evaluate their influence during the construction of ROC curves for WC cut-off point selection.

## 2. Research Design and Methods

### 2.1. Subject Selection

The Maracaibo City Metabolic Syndrome Prevalence Study (MMSPS) [14] was a cross-sectional research study undertaken in the city of Maracaibo-Venezuela, whose purpose was the identification and analysis of MS and cardiovascular risk factors in the adult population of Maracaibo, the second largest city in Venezuela with 2.750.00 inhabitants. The methodology and randomization during sampling were published elsewhere [14]. Currently, there are 2,230 subjects enrolled [14], out of which 1,902 were selected, therefore excluding those individuals whose serum insulin levels were not determined and those diagnosis with diabetes mellitus; the latter was excluded because pharmacological treatment of these patients would modify the variables used in this research. The study was approved by the Bioethics Committee of the Endocrine and Metabolic Diseases Research Center, University of Zulia, and all participants signed a written consent before being interrogated and physically examined by a trained team.

### 2.2. Clinical Evaluation

The assessment of blood pressure was done applying the auscultatory technique, and HBP classification was made using the criteria proposed in the VII Joint National Committee on Prevention, Detection, Evaluation, and Treatment of High Blood Pressure [15]. Mean Arterial Pressure (MAP) [16] was calculated using the equation (Diastolic Pressure + (Systolic Pressure − Diastolic Pressure/3)), expressed in mmHg. Obesity was classified applying the WHO criteria [17] based on the BMI value. Weight was assessed using a digital scale (Tanita, TBF-310 GS Body Composition Analyzer, Tokyo, Japan), while Height was obtained with a calibrated rod, with the patients shoeless and wearing light clothing. WC was measured using calibrated measuring tape in accordance to the anatomical landmarks proposed by the USA National Institutes of Health protocol [18].

### 2.3. Biochemical Analyses

Fasting levels of glucose, cholesterol, triglycerides (TAG), HDL-C, and hs-CRP were determined using an automatized computer analyzer (Human Gesellschaft für Biochemica und Diagnostica mbH). LDL and VLDL levels were calculated applying the Friedewald formulas [19]. When triacylglycerides were over 400 mg/dL measurement was done using lipoprotein electrophoresis and optical densitometry (BioRad GS-800 densitometer, USA). Insulin was determined using an ultrasensitive ELISA method (DRG Instruments GmbH, Germany, International DRG Division, Inc). The MS diagnosis was done using the IDF/NHLBI/AHA-2009 consensus criteria [20].

### 2.4. Insulin Sensitivity

This was assessed by the Homeostasis Model Assessment (HOMA2-IR) calculator, which is available at http://www.dtu.ox.ac.uk/homacalculator/index.php from the Oxford Centre for Diabetes, Endocrinology and Metabolism. Using ROC curve construction technique, our research team determined that the optimal cutpoint for HOMA2-IR for our population is 2.00 [21].

### 2.5. Statistical Analysis

Database construction and cluster analysis were done using the Statistical Package for the Social Sciences (SPSS) v19 for Windows (IBM Inc., Chicago, IL), while the ROC curves were constructed using the R Project for Statistical Computing, available at http://www.r-project.org/. Normal distribution of continuous variables was assessed using Geary's test; for normally distributed variables, the results were expressed as arithmetic mean ± SD (standard deviation). Variables without normal distribution were logarithmically transformed, and normal distribution subsequently corroborated. The differences between arithmetic means were assessed using Student's *t*-test (when two groups were compared) or one-way ANOVA (when three or more groups were compared). Qualitative variables were expressed as absolute and relative frequencies.

#### 2.5.1. Cluster Analysis Protocol

Previously to the Two-Step Cluster Analysis, all individuals were classified according to BMI in Normal Weight, Overweight, and Obese. The obese groups were primarily evaluated as a group (Obese, BMI ≥ 30 kg/m^2^) and according to WHO classification (Class I, Class II, and Class III) [17]; since the results showed similar behavior between them, we decided to use the classification of Obesity because it allowed us to evaluate the subjects more clearly.

**Figure 1:**
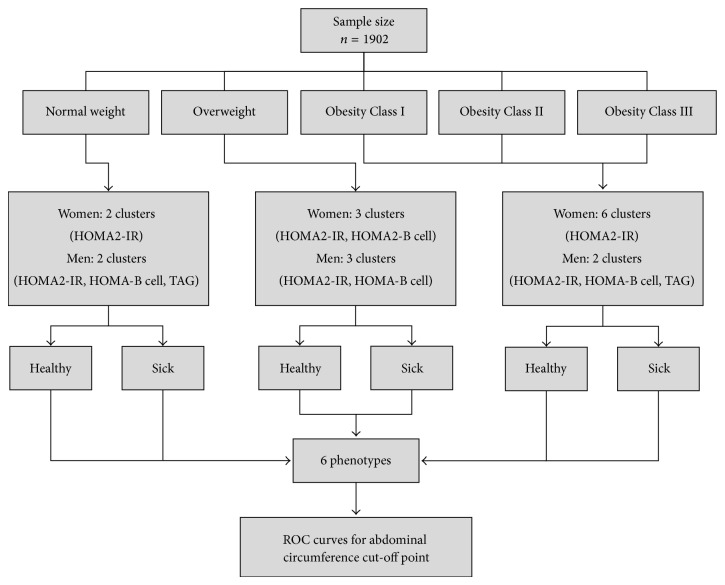
Diagramshowing the two-stage clustering method to properly categorize the subjects into healthy and sick groups according to the selected predictors.

Each BMI category was submitted independently to the cluster analysis, categorizing the subjects as metabolically healthy or sick; see Figure 1. The metabolic variables evaluated as possible metabolic predictors based on their physiological function and biological plausibility were MAP, TAG, total cholesterol, HDL-C, HOMA2-IR, HOMA2-*β*cell, HOMA2-S, fasting blood glucose, non-HDL-C cholesterol, TAG/HDL-C index, and hs-CRP; WC was excluded because it was the assessed dependent variable. The predictive strength of these variables was analyzed in accordance to cluster ability and quality, ranging from 0.0 to 1.0. The best metabolic predictive variables selected were (a) HOMA2-IR and HOMA2-*β*cell for normal weight women; (b) HOMA2-IR, HOMA2-*β*cell and TAG for normal weight men; (c) HOMA2-IR and HOMA2-*β*cell for overweight women; (d) HOMA2-IR, HOMA2-*β*cell, and TAG for overweight men; and (e) HOMA2-IR for male and female obese patients (Table 1).

The Two-Step Cluster Analysis for SPSS was conducted in two phases [22]: during the first step (called “precluster”), the subjects are divided into several small subclusters. Then, the obtained subclusters are grouped into a preferred number of clusters; if the desired number of clusters is unknown, the SPSS Two-Step Cluster Component will find the proper number of clusters automatically. Once the program analyzed the subclusters and the characteristics of each BMI category (as described previously), the subjects were categorized in 6 phenotypes: HNW, MONW, healthy and metabolically disturbed overweight, MDO, and MHO.

#### 2.5.2. Cluster Quality Measures

To evaluate the quality of the resulting clusters, the cohesion, separation, and silhouette coefficient were calculated [23–25]. The silhouette coefficient [26] encompasses the ideas of cohesion (the closeness of related objects in a cluster) and separation (the distance between objects in a cluster), describing the average distances between variables within a cluster and between other clusters, the highest silhouette results being between 0.5 and 1 [23, 26]. The clusters with high cohesion are preferred because it is a guarantee of good quality clustering, demonstrated by high silhouette values and truly clustered variables [23–26].

#### 2.5.3. Cross-Validation Technique

Cluster validation aims to evaluate the differences within a cluster in order to confirm clustering selection to estimate the accuracy of a prediction model [27]. This method requires the division of the data into two groups: one to* train* (training dataset) and the other to* validate *(testing dataset) [27, 28]. The process requires doing several rounds of partitioning and cross-validation, where all the analyses are performed on the training set and then validating such analyses in the testing set [27, 28], and agreement was assessed by Cohen's kappa coefficient.

#### 2.5.4. ROC Curves Construction

**Figure 2:**
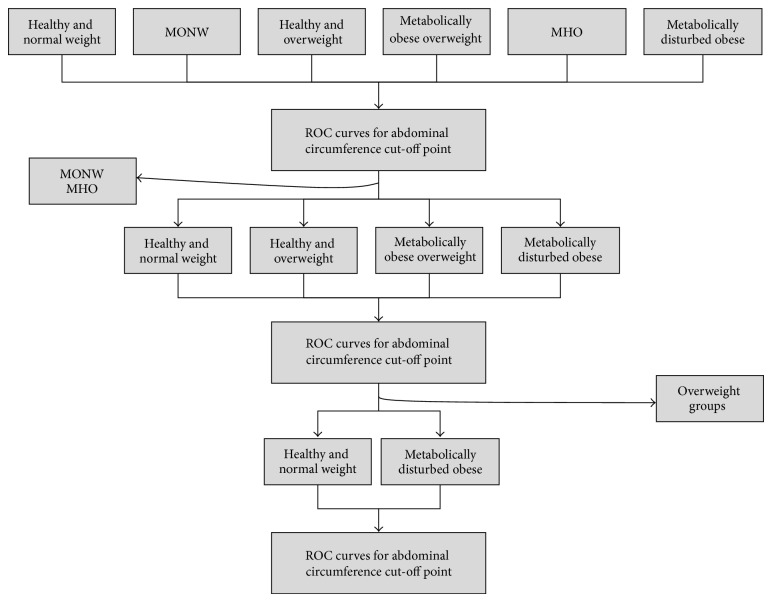
Diagram that depicting the selection process during the phenotype analysis and exclusion to determine the proper cut-off point for WC.

The Receiving Operating Characteristic (ROC) [29] curves were used to analyze the predictive validity and to determine optimal cut-off values for WC following a series of exclusion steps (Figure 2). Comparison of AUC was calculated with DeLong's Test [30]. Several indexes were calculated to assess the optimal cut-off point on the curve, such as the Youden Index, the distance of the point closest to (0.1) on the ROC curve and Positive Likelihood Ratio were calculated [31]. Nevertheless, sensitivity over specificity was considered when selecting WC cut-off points.

## 3. Results

### 3.1. General Characteristics of the Population

An overall 1,902 subjects were studied, out of which 52.15% were women and 47.84% were men. Age arithmetic mean for all participants was 38.70 ± 15.06 years (IC 95%, 38.02–39.08), 37.17 ± 14.54 years for men (IC 95%, 36.85–38.65) and 40.11 ± 15.29 years for women (IC 95%, 39.15–41.06). Distribution of the population according to age groups, ethnic groups, BMI, and MS diagnosis is shown in Table 2. For anthropometric parameters, biochemical, and blood pressure results see Table 3.

### 3.2. Two Steps Cluster Analysis

Using all the information obtained from the clusters, six phenotypes were generated: HNW (28.29%), MONW (3.36%), Healthy Overweight (28.08%), Metabolically disturbed Overweight (7.47%), MHO (11.20%), and MDO (21.60%) (Table 5); note that MONW and MHO subjects represent 14.56% of the total sample.  Table 6 shows general biochemical characteristics of the 6 phenotypes built by cluster analysis.

Cluster quality was assessed with the silhouette coefficient, which rendered >0.5 for every cluster, meaning that all clusters were classified as good models. Next, cross-validation was performed, dividing the subjects in two groups: S1 and S2. The S1 group was used as the training set, where centroid-based clustering was calculated using the steps shown in Figure 1. The S2 group was used as the validating set (S2), where clusters were obtained using two methods: (a) normal clustering process (S2-clusters) and (b) clustering based on centroids and distances obtained from S1 (S2 clusters according to S1). All the resulting S2-derived clusters were compared using Cohen's kappa, resulting in 0.902; *P* < 0,00001 (Table 4).

### 3.3. ROC Curves

#### 3.3.1. Curves Constructed with the Overall Population (All 6 Groups)

**Figure 3:**
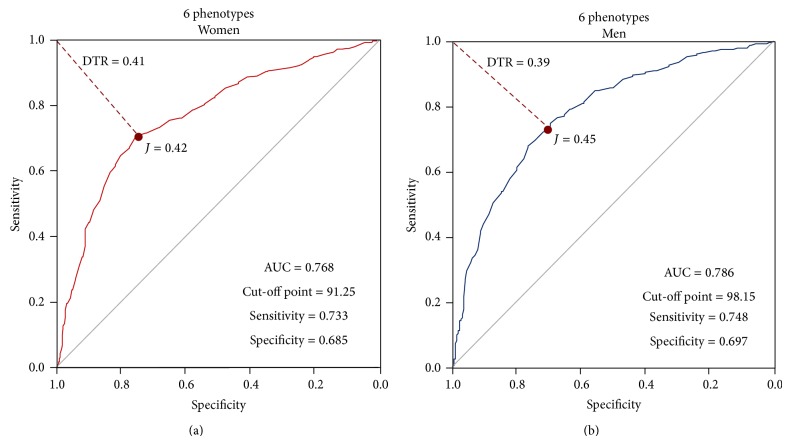
ROC curves constructed using 6 phenotypes.

We sought to find an appropriate cut-off point for this population sample, applying the 6 phenotypes previously described in a stepwise manner. In Figure 3, ROC curves for men and women are shown. In Figure 3(a), the selected cut-off point for women was 91.25 cm, with an AUC 0.768, sensitivity of 73.3%, and a specificity of 68.5% (Table 7). In the next panel, Figure 3(b), the chosen cut-off point for men was 98.15 cm, with an AUC of 0.786, 74.8% sensitivity, and 69.7% specificity.

#### 3.3.2. COR Curves Construction without MONW and MHO Groups (4 Groups)

**Figure 4:**
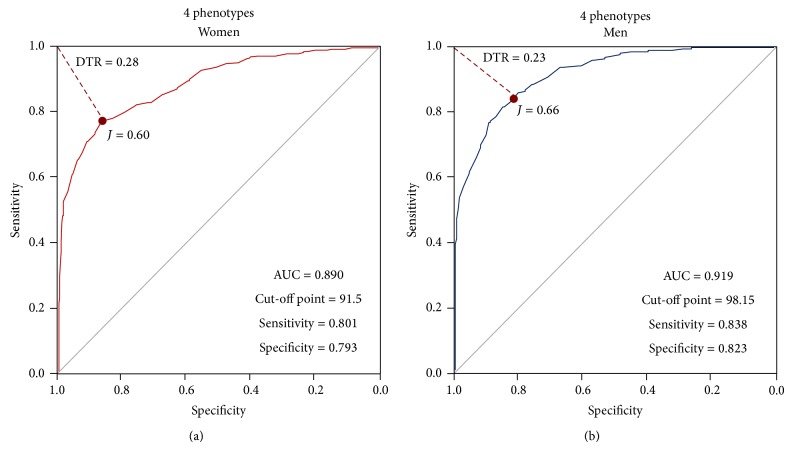
ROC curves constructed using 4 phenotypes, after the exclusion of MOWN and MHO groups.

The following ROC curves were built without the “anomalous signals” derived from the atypical phenotypes, MONW and MHO. In Figure 4(a), the women's ROC curve is depicted, with a selected cut-off point of 91.50 cm, showing an AUC of 0.890, 80.1% sensitivity, and 79.3% specificity. In the following panel, Figure 4(b), the selected cut-off point for men was 98.15 cm, with an AUC of 0.919, sensitivity of 83.8%, and a specificity of 82.3% (Table 7).

#### 3.3.3. COR Curves Construction Excluding MONW, MHO, and Overweight Groups (2 Groups)

**Figure 5:**
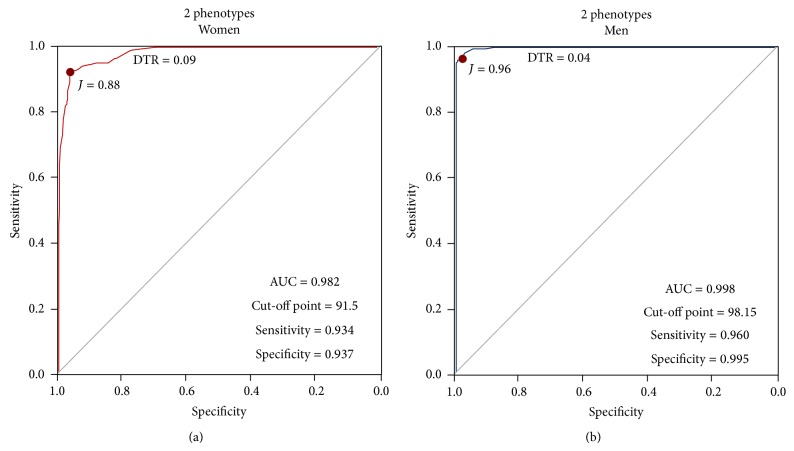
ROC curves constructed using 2 phenotypes, after exclusion of Overweight groups.

**Figure 6:**
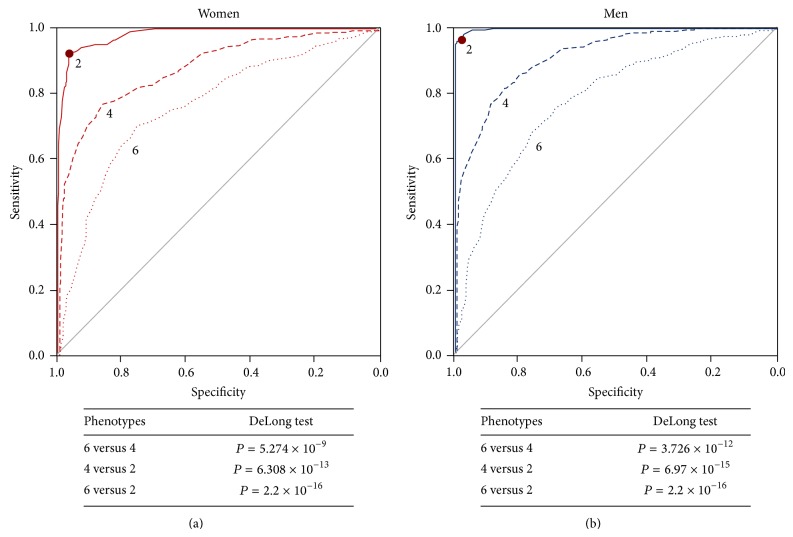
Comparison of the ROC curves constructed in the overall stepwise process for both men and women. Notice that the curves get closer to the 1.0 corner as the true healthy and sick subjects are being selected. The DeLong test results are shown as well.

The final ROC curves were built without the Overweight groups, leaving only the HNW and the MDO. In Figure 5(a), the women's ROC curve is shown, with a chosen cut-off point of 91.5 cm, characterized by an AUC of 0.982, sensitivity of 93.4%, and a specificity of 93.7%. In Figure 5(b), the men's cut-off point was 98.15 cm, with an AUC of 0.998, sensitivity of 96%, and a specificity of 99.5%; see Table 7.  Figure 6 shows all the constructed ROC curves and their DeLong results. Finally, Table 8 shows the metabolic variables of the subjects categorized with the obtained WC cut-off points from this investigation, resulting in significant differences between obese and nonobese subjects in every variable, except in HOMA-2*β*cell in the women's group.

## 4. Discussion

It is imperative to determine accurate WC cut-off values in order to diagnose abdominal obesity, given the progressive and fast rise in the worldwide prevalence of this disease. This growing epidemic has been a driving force for the development of improved diagnostic tests to aid physicians in their daily practice to diagnose abdominal obesity associated with metabolic disorders [31]. The search for ethnic-specific values for anthropometric measures requires the application of several techniques, ROC curves being one of the tools available in order to ascertain an appropriate cut-off point [32].

ROC curves approach to determine suitable cut-off points for WC has been extensively used [9–11], especially in populations that are not properly classified in the latest MS criteria by the IDF/NHLBI/AHA-2009 due to lack of sufficient population-specific data for the WC variable [20]. As opposed to more widespread methodology in these studies [9], the involvement of data mining techniques (cluster analysis) enhances the selection of healthy and sick subjects for the construction of ROC curves because it does not use predetermined variables nor arbitrary cut-off points to decide; instead it allows the program to group the individuals according to their biological characteristics and spontaneous tendencies [22]. This improvement in subject classification guarantees cutoff points with superior sensitivity and specificity, which is the ultimate goal in surveys such as ours.

Several studies have suggested that the WC cut-off proposed by the IDF/NHLBI/AHA-2009 consensus seemed to be invalid for certain ethnicities, particularly the Hispanic groups in Latin America [9]. Aschner et al. [10] published their WC cut-off points based on ROC curves using visceral fat area (≤100 cm^2^) as the independent variable, with resulting cut-off values of 94 cm for men (89.9% sensitivity and 80.2% specificity) and 90–92 cm for women (78.9%–72.9% sensitivity and 67.6%–74.5% specificity). However, Aschner's research conveys the use of visceral fat to find an optimal cut-off value of WC which detects subjects at risk of abdominal obesity. A cut-off point of 100 cm^2^ was calculated for Japanese population [33], using metabolic criteria cutoffs that are now considered outdated (e.g., fasting glucose >110 mg/dL). Moreover, Latin-Americans are phenotypically and genetically different from Asians [34], which hinders the possibility of properly extrapolating results from their group onto ours. Despite these shortcomings, this cut-off has been used in several studies as a standard. Currently, Latin America also needs cut-off values concerning visceral fat, especially when ethnic minority groups are included, such as the Amerindians and Afro-Descendants.

Two-Step Cluster Analysis approach enhances sorting of the subjects, allowing for better grouping and evaluation according to biochemical and anthropometric coalescent variables, eliminating the bias observed in predetermined variables and cut-off points. On this reasoning, 6 phenotypes were constructed: Healthy Normal-Weight, MONW, Healthy and Metabolically Disturbed Overweight, MHO, and Metabolically Disturbed Obese. Each group has diverse cardiometabolic profiles which have been widely described in the last decade [5–8, 13]. Evidently, the MONW and MHO are exceptions to rules that have been described traditionally, where first glance examination of an obese or lean patient would automatically classify them as sick or healthy, respectively. Using the selected parameters according to BMI, a proper classification is possible, being demonstrated by the enhancement of sensitivity, specificity, and AUC for abdominal circumference in these groups.

ROC curve programs allow the determination of true positive and negative cases, by providing cut-off points and their corresponding AUC, sensitivity, and specificity; nevertheless, this feature depends on an appropriate sorting of the sample and its accuracy is confirmed with the comparison of curves before and after selection. Eliminating noise during the filtering of information is of paramount importance, since it behaves as phantom signals which derail the evaluation towards inaccurate values. Exclusion of the MONW and MHO categories impedes the use of false data to determine a cut-off point, rendering enough sensitivity and specificity to identify subjects at risk. It is imperative that physicians embrace the advantages offered by both techniques in order to be able to determine valid cutoff points in ethnic-based studies concerning metabolic variables, which are categorized as biological and thus display a continuous behavior.

The other groups that got excluded were the Overweight individuals. The definition of overweight lies between normalcy and obesity, between 25.00 and 29.99 kg/m^2^. This allocation confers this definition a “transition” quality which is based on the possibility of reducing weight and achieving normal weight or augmenting weight reaching obesity levels [35, 36]. Moreover, this also suggests that weight is a continuous biological factor, and the arbitrary classification of overweight is a transition phase in the natural history of obesity [36]. Therefore, since overweight subjects and considered “in transition” were removed them from the final WC ROC curve construction.

It is necessary to emphasize three facts: WC alone cannot recognize MONW or MHO subjects, anthropometry is insufficient to determine healthiness, and relying on the information obtained in other variables is needed for the filtering process. These facts open a new window of opportunity to investigate the establishment of new strategies that can help identify peculiar phenotypes, such as the use of somatotypes constructed with local anthropometric and biochemical data, facilitating the identification of MOWN and MHO subjects.

The chosen cut-off point for the Women's group was 91.25 cm, very similar to that reported by Herrera et al. [9], suggesting that the females in the sample tend to have higher WC values than the ones set previously both by ATPIII [37] (<88 cm) and IDF/NHLBI/AHA-2009 (<80 cm). This finding is probably explained by differences regarding height, fat distribution, and genetic background [38]. It is noteworthy to point out that, even after the groups were filtered extracting MOWN, MHO, and overweight individuals, the WC cut off point always remained the same, but sensitivity and specificity improved significantly, proving that this approach offers a better way to scrutinize metabolically heterogeneous groups. Women appear to boast higher WC cut-offs, perhaps due to displaying a central fat distribution despite having femoral-gluteal fat distribution tendencies. Regarding males, a similar trend was observed, this time with a selected cut-off of 98.15 cm which is between the cutoff points proposed in IDF/NHLBI/AHA-2009 (<90 cm) and ATPIII (<102 cm).

We have previously published the prevalence of obesity in the city of Maracaibo [1], reporting that the overall prevalence of abdominal obesity using IDF/NHLBI/AHA-2009 criteria [20] was 74.2%, while using the ATPIII criteria [37] rendered a prevalence of 51.7%. Using the cut-off points proposed in this research, the overall abdominal obesity prevalence using the complete sample of MMSPS (*n* = 2,230) is 35.6% (*n* = 794). Thus, the new WC cut-off point reduces the alarming 74.2% obtained with the harmonizing criteria and offers better information to design strategies for primary and secondary prevention.

Lastly, we address two important limitations within this investigation. First, the absence of imaging study confirmation such as visceral fat measurement; this branch of the MMSPS study is currently underway. Second, we used BMI as a method of diagnosis and categorization instead of other obesity diagnostic tools like DEXA for Body Composition [39] due to lack of resources for such endeavor. It has been reported that BMI has limitations in regard to adiposity diagnosis, especially in intermediate ranges of BMI [40]. However, BMI cutoff point of ≥30 kg/m^2^ should be easily dismissed, since it has been associated with high specificity and positive predictive value for diagnosing obesity in both sexes [40] and strong association with other entities such as arterial hypertension [41], diabetes mellitus [42], stroke [43], premature death [44], and several types of cancer [45].

In conclusion, we propose WC cut-off points of <91.00 cm for women and <98.00 cm for men, both providing an excellent sensitivity and specificity when concerning the diagnosis of abdominal obesity. The need for ethnic-specific WC cut-off points is paramount, especially when there is an association between WC and mortality prediction [46, 47]. The application of statistical methods that allow the filtering and gathering of accurate information, like Cluster Analysis and ROC curve constructs, will warrantee production of veracious cut-off points that can be applied in large prospective trials.

## Figures and Tables

**Table 1:** Risk factors aggregation according to Cluster Analysis and derivatives clusters. Maracaibo City Metabolic Syndrome Prevalence Syndrome, 2013.

Phenotypes	Cluster 1	Cluster 2	Cluster 3	Cluster 4	Cluster 5	Cluster 6
Normal-weight females	Size: 7.7% **HOMA2-IR** = 3.56	Size: 92.7 **HOMA2-IR** = 1.47^*^				

Normal-weight males	Size: 15.1% **HOMA2-IR** = 2.54 **HOMA2-Bcell** = 172.82 **TAG = **180.99	Size: 84.9% **HOMA2-IR** = 1.15 **HOMA2-Bcell** = 109.69 **TAG = **84.65^*^				

Overweight females	Size: 24.1% **HOMA2-IR** = 3.12 **HOMA2-Bcell** = 195.18	Size: 25.3% **HOMA2-IR** = 0.97 **HOMA2-Bcell** = 85.14^*^	Size: 50.6% **HOMA2-IR** = 1.69 **HOMA2-Bcell** = 137.06^*^			

Overweight males	Size: 18.3% **HOMA2-IR** = 3.57 **HOMA2-Bcell** = 212.77	Size: 46.1% **HOMA2-IR** = 1.80 **HOMA2-Bcell** = 144.05^*^	Size: 35.6% **HOMA2-IR** = 1.09 **HOMA2-Bcell** = 90.33^*^			

Obese Class I females	Size: 1.1% **HOMA2-IR** = 10.30	Size: 4.8% **HOMA2-IR** = 5.12	Size: 9.0% **HOMA2-IR** = 3.95	Size: 31.4 % **HOMA2-IR** = 1.16^*^	Size: 35.1% **HOMA2-IR** = 2.06	Size: 18.6% **HOMA2-IR** = 2.86

Obese Class II females	Size: 1.2% **HOMA2-IR** = 8.60	Size: 2.4% **HOMA2-IR** = 8.40	Size: 3.6% **HOMA2-IR** = 6.37	Size: 34.1% **HOMA2-IR** = 1.38^*^	Size: 32.9% **HOMA2-IR** = 2.52	Size: 25.6% **HOMA2-IR** = 3.66

Obese Class III females	Size: 4.8% **HOMA2-IR** = 1.31	Size: 14.3% **HOMA2-IR** = 4.5	Size: 2.4% **HOMA2-IR** = 9.0	Size: 14.3% **HOMA2-IR** = 3.67	Size: 31.0% **HOMA2-IR** = 2.35	Size: 33.3 % **HOMA2-IR** = 1.31^*^

Obese Class I male	Size: 1.0% **HOMA2-IR** = 8.60	Size: 11.2% **HOMA2-IR** = 4.60	Size: 19.8% **HOMA2-IR** = 3.11	Size: 28.3% **HOMA2-IR** = 2.19	Size: 26.3% **HOMA2-IR** = 1.59^*^	Size: 13.6% **HOMA2-IR** = 0.93^*^

Obese Class II male	Size: 2.7% **HOMA2-IR** = 8.10	Size: 16.0% **HOMA2-IR** = 3.36	Size: 26.7% **HOMA2-IR** = 3.36	Size: 28.0% **HOMA2-IR** = 1.71^*^	Size: 17.3% **HOMA2-IR** = 2.45	Size: 9.3% **HOMA2-IR** = 0.70^*^

Obese Class III male	Size: 2.6% **HOMA2-IR** = 10.10	Size: 10.3% **HOMA2-IR** = 7.02	Size: 15.4% **HOMA2-IR** = 5.83	Size: 20.4% **HOMA2-IR** = 4.11	Size: 38.5% **HOMA2-IR** = 2.78	Size: 12-8% **HOMA2-IR** = 1.52^*^

The cells with “∗” indicate the “healthy” clusters for each phenotype. The cells without “∗” represent the “sick” clusters of persons.

HOMA2-IR: Homeostasis Model Assessment-2 for Insulin Resistance; HOMA2-Bcell: Homeostasis Model Assessment-2 for Pancreatic *β* Cell Function; TAG: triglycerides, expressed in mg/dL.

**Table 2:** General characteristics of the population from Maracaibo City Metabolic Syndrome Prevalence Study, 2013.

	Women		Men		Total	
	*n*	%	*n*	%	*n*	%
Age groups						
18-19	87	8.8	70	7.7	157	8.3
20–29	218	22.0	294	32.3	512	26.9
30–39	176	17.7	178	19.6	354	18.6
40–49	230	23.2	164	18.0	394	20.7
50–59	170	17.1	136	14.9	306	16.1
60–69	74	7.5	46	5.1	120	6.3
70 and more	37	3.7	22	2.4	59	3.1
Ethnic groups						
Mixed race	757	76.3	706	77.6	1463	76.9
Hispanic White	162	16.3	138	15.2	300	15.8
Afro-Venezuelan	24	2.4	31	3.4	55	2.9
Amerindian	39	3.9	34	3.7	73	3.8
Other	10	1.0	1	0.1	11	0.6
BMI						
Low weight	29	2.9	11	1.2	40	2.1
Normal weight	335	33.8	227	24.9	562	29.5
Overweight	316	31.9	360	39.6	676	35.5
Obesity grade I	188	19.0	198	21.8	386	20.3
Obesity grade II	82	8.3	75	8.2	157	8.3
Obesity grade III	42	4.2	39	4.3	81	4.3
MS IDF/NHLBI/AHA-2009						
Absence	627	63.2	520	57.1	1147	60.3
Presence	365	36.8	390	42.9	755	39.7
Total	**992**	**100.0 **	**910**	**100.0 **	**1902**	**100.0 **

**Table 3:** General characteristics of the population according to gender. Maracaibo City Metabolic Syndrome Prevalence Study, 2013.

	Women		Men		Total	
	Mean	SD	Mean	SD	Mean	SD

BMI	27.92	6.25	28.77	6.21	28.32	6.24
WC	91.12	13.96	98.40	15.85	94.57	15.32
FG	98.20	31.01	99.09	32.73	98.62	31.84
Insulin	14.57	9.34	14.83	9.83	14.69	9.58
HOMA2-IR	2.03	1.29	2.08	1.37	2.05	1.33
HOMA2-B cell	136.97	58.67	137.25	65.83	137.10	62.19
HOMA2-S	66.70	42.35	69.11	45.47	67.85	43.88
T-Chol	193.77	44.41	187.32	47.33	190.71	45.92
TAG	117.52	87.44	144.00	114.79	130.08	102.18
HDL-C	46.90	11.89	40.95	11.42	44.08	12.04
SBP	117.69	17.29	121.85	15.98	119.66	16.80
DBP	75.65	10.81	79.03	11.46	77.25	11.25
MAP	89.66	12.17	93.30	12.12	91.39	12.28
Non-HDL-C	146.87	45.08	146.37	47.76	146.63	46.36
TAG/HDL	2.86	3.03	4.03	4.27	3.41	3.72

**Table 4:** Distribution of the subjects (training and testing datasets) during cross-validation process.

	Cluster Analysis in S2							
	Cluster 1		Cluster 2		Cluster 3		Total	
	*n*	%	*n*	%	*n*	%	*n*	%

Cluster Analysis in S2 according to S1								
Cluster 1	609	64.0	0	0	31	3.3	**640 **	**67.3 **
Cluster 2	0	0	50	5.3	0	0	**50 **	**5.3 **
Cluster 3	0	0	14	1.5	247	26.0	**261 **	**27.4 **
Total	**609 **	**64.0 **	**64 **	**6.7 **	**278 **	**29.2 **	**951 **	**100.0 **

S1: a subgroup of subjects randomly selected from the database.

S2: a subgroup of subjects randomly selected from the database.

Cohen's kappa coefficient: 0.902 (*P* < 0.00001).

**Table 5:** Distribution of the population according to BMI and Metabolic Health Status. Maracaibo City Metabolic Syndrome Prevalence Syndrome, 2013.

	Women						Men					
	Healthy		Sick^*^		Total		Healthy		Sick^*^		Total	
	n	%	n	%	N	%	n	%	n	%	n	%
Normal weight	336	92.3	28	7.7	364	100.0	202	84.9	36	15.1	238	100.0
Overweight	240	75.9	76	24.1	316	100.0	294	81.7	66	18.3	360	100.0
Obesity class 1	59	31.4	129	68.6	188	100.0	79	39.9	119	60.1	198	100.0
Obesity class II	28	34.1	54	65.9	82	100.0	28	37.3	47	62.7	75	100.0
Obesity class III	14	33.3	28	66.7	42	100.0	5	12.8	34	87.2	39	100.0
Total	**677**	**68.2 **	**315**	**31.8 **	**992**	**100.0 **	**608**	**66.8 **	**302**	**33.2 **	**910**	**100.0 **

^*^The “Sick” term applied here conveys the metabolically disturbed subjects which have metabolic obese profiles.

**Table 6:** Metabolic variables behavior of each metabolic phenotype according to sex. Maracaibo City Metabolic Syndrome Prevalence Syndrome, 2013.

	Women		Men	
	Mean	SD	Mean	SD
WC				
HNW	79.27	8.24	81.54	6.87
MONW	77.20	7.07	86.98	7.62
Healthy overweight	89.69	6.88	94.82	6.59
Sick^*^ overweight	90.27	7.59	97.76	6.10
MHO	104.40	10.55	109.17	11.94
MDO	105.50	10.07	116.04	15.32
HOMA2-IR				
HNW	1.47	0.48	1.15	0.44
MONW	3.56	1.36	2.54	1.47
Healthy overweight	1.45	0.51	1.49	0.58
Sick^*^ overweight	3.12	1.14	3.57	1.41
MHO	1.24	0.37	1.40	0.41
MDO	3.14	1.54	3.39	1.42
TAG				
HNW	86.49	52.06	84.65	38.44
MONW	120.61	155.70	180.99	124.76
Healthy overweight	112.97	68.92	131.01	80.97
Sick^*^ overweight	131.32	93.03	173.92	106.29
MHO	120.12	73.96	143.08	118.27
MDO	144.57	95.54	184.60	121.59
HDL-C				
HNW	49.29	11.81	46.00	11.16
MONW	51.61	11.54	39.50	11.84
Healthy overweight	48.17	11.65	41.91	12.88
Sick^*^ overweight	44.43	10.84	38.45	8.39
MHO	45.57	13.01	40.15	9.91
MDO	44.13	11.45	36.71	8.48
MAP				
HNW	83.80	9.60	87.28	9.92
MONW	87.00	13.17	88.28	10.54
Healthy overweight	89.20	11.33	93.44	11.77
Sick^*^ overweight	88.85	10.46	91.45	10.39
MHO	95.78	13.91	96.38	13.17
MDO	95.10	12.07	98.01	12.47

^*^The “Sick” term applied here conveys the metabolically disturbed subjects which have metabolically obese profiles.

HDL-C, high density lipoprotein, expressed in mg/dl; HOMA2-IR: Homeostasis Model Assessment-2 for Insulin Resistance; MAP: mean arterial pressure, expressed in mmHg; TAG: triglycerides, expressed in mg/dL; WC, waist circumference, expressed in cm.

**Table 7:** Waist circumference cut-offs based on ROC Curves. Sensitivity, Specificity, Youden's Index, Positive Likelihood, and Distance to the ROC Curve. Maracaibo City Metabolic Syndrome Prevalence Syndrome, 2013.

	WC (cm)	Sensitivity (%)	Specificity (%)	Youden's Index	Distance to ROC	LR+
6 phenotypes						
Women	90.75	75.2	65.3	0.41	0.42	2.16
	91.25^¶^	73.3	68.5	0.42^Ψ^	0.41^§^	2.32
	91.75	73.3	68.4	0.42^Ψ^	0.41^§^	2.32
Men	97.75	76.5	67.8	0.44	0.40	2.37
	98.15^¶^	74.8	69.7	0.45^Ψ^	0.39^§^	2.46
	98.40	74.5	69.7	0.44	0.40	2.45

4 phenotypes						
Women	90.75	82.2	75.9	0.58	0.29	3.41
	91.5^¶^	80.1	79.3	0.60	0.28^§^	3.86
	92.25	78.4	83.0	0.61^Ψ^	0.28^§^	4.61
Men	97.50	85.7	80.2	0.66^Ψ^	0.24	4.32
	98.15^¶^	83.8	82.3	0.66^Ψ^	0.23^§^	4.73
	98.40	83.5	82.3	0.66^Ψ^	0.24	4.71

2 phenotypes						
Women	90.75	94.3	92.6	0.87	0.09^§^	12.74
	91.5^¶^	93.4	93.7	0.88^Ψ^	0.09^§^	14.82
	92.5	92.9	94.6	0.88^Ψ^	0.09^§^	17.20
Men	97.00	96.0	98.5	0.95	0.04^§^	64.00
	98.15^¶^	96.0	99.5	0.96^Ψ^	0.04^§^	192.00
	98.65	95.5	99.5	0.95	0.05	191.00

6 phenotypes: HNW, MONW, MHO, MDO, and Overweight healthy and sick groups

4 phenotypes: HNW, MONW, and Overweight healthy and sick groups.

2 phenotypes: HNW and MDO.

(¶) Selected cut-off (in cm) based on Sensitivity, Specificity, Youden Index, and Positive Likelihood Ratios (LR+), giving emphasis to highest sensitivity values.

(Ψ) Cutpoint 1, asserted using the maximum Youden Index.

(§) Cutpoint 2, obtained from the point closet to the ROC (0.1).

**Table 8:** General characteristics of the subjects after categorization using the newly selected WC cutpoints. Maracaibo City Metabolic Syndrome Prevalence Study, 2013.

	Women					Men				
	Abdominal obesity^*^				*P*	Abdominal obesity^**^				*P*
	Absence		Presence			Absence		Presence		
	Mean	SD	Mean	SD		Mean	SD	Mean	SD	
BMI (Kg/m^2^)	23.84	3.33	32.58	5.58	<0.0001	24.98	3.22	33.30	6.03	<0.0001
WC (cm)	80.84	7.37	102.85	9.26	<0.0001	87.22	7.47	111.60	12.94	<0.0001
FG (mg/dL)	12.67	6.96	16.62	11.04	<0.0001	11.37	7.16	18.66	11.04	<0.0001
Insulin (*µ*UI/mL)	90.29	11.67	98.28	21.81	<0.0001	89.56	12.18	99.99	28.10	<0.0001
HOMA2-IR	146.16	55.94	150.01	62.82	<0.0001	135.45	50.81	165.07	80.20	<0.0001
HOMA2-Bcell	67.59	39.68	56.62	37.36	0.313	79.18	43.15	49.25	35.45	<0.0001
HOMA2-S	1.85	0.96	2.46	1.55	<0.0001	1.67	1.03	2.77	1.57	<0.0001
T-Chol (mg/dL)	185.04	41.95	204.04	45.07	<0.0001	180.75	42.54	196.06	51.89	<0.0001
TAG (mg/dL)	93.86	66.33	136.68	87.99	<0.0001	113.00	73.17	170.87	118.50	<0.0001
HDL-c (mg/dL)	48.80	11.77	45.32	11.86	<0.0001	43.55	12.65	38.14	8.78	<0.0001
SBP (mmHg)	112.12	14.47	122.97	18.34	<0.0001	117.35	14.25	126.74	16.35	<0.0001
DBP (mmHg)	72.03	9.48	79.36	10.99	<0.0001	75.73	10.52	82.65	11.63	<0.0001
MAP (mmHg)	85.40	10.43	93.90	12.46	<0.0001	89.61	10.92	97.34	12.35	<0.0001
Non-HDL-c (mg/dL)	136.24	42.45	158.71	45.09	<0.0001	137.20	42.99	157.92	51.51	<0.0001
TAG/HDL	2.17	2.14	3.38	2.94	<0.0001	2.92	2.41	4.92	4.06	<0.0001

BMI, body mass index; FG, fasting glycemia; HDL-c, high density lipoprotein; MAP, mean arterial pressure; SBP, systolic blood pressure; DBP, diastolic blood pressure; T-Chol, total cholesterol; TAG, triglycerides; TAG/HDL, TAG/HDL index; WC, waist circumference, expressed in cm. ^*^WC ≥ 91.50 cm; ^**^WC ≥ 98.15 cm.
